# Self-Monitoring Accuracy over Time for a Complex Diagnostic Skill

**DOI:** 10.5334/pme.1767

**Published:** 2026-05-14

**Authors:** Wolf E. Hautz, Thimo Marcin, Stefan K. Schauber, Robin Walter, Stefanie C. Hautz, Tanja Birrenbach, Beat Lehmann, Thomas C. Sauter, Roman Hari, Juliane E. Kämmer

**Affiliations:** 1 Department of Emergency Medicine, Inselspital University Hospital Bern, University of Bern, Bern, Switzerland; 2 Centre for Educational Measurement, Faculty of Educational Sciences and the Centre for Health Sciences Education, Faculty of Medicine, University of Oslo, Oslo, Norway; 3 Institute of Primary Health Care (BIHAM), University of Bern, Bern, Switzerland

## Abstract

**Background:**

Moment-to-moment self-monitoring correlates well with task performance. However, little is known about the development of self-monitoring accuracy over time. This retention study explores the long-term development of self-monitoring in the performance of a complex clinical task.

**Methods:**

Advanced medical students, without prior ultrasound skills, participated in an ultrasound course and were evaluated using OSCEs immediately after the course (T1) and after a six-month follow-up (T2). Assessment included expert evaluation of skills and self-reported confidence levels at each station. Two linear mixed models were used to track changes of performance and confidence over time, and assess the effect of additional training, demographics, and performance on confidence levels.

**Results:**

The study included 141 medical students (65% female, median age 22 years). Performance scores across six OSCE stations were significantly higher at T1 compared to T2, with median scores of 36.0 and 34.3 points (on a scale of 0–50), while confidence levels decreased from a median of 3 to 2.8 (on a scale of 1–5). On average, self-reported confidence decreased in some proportion to the skill decline over the follow-up period of six months, although individual students displayed various patterns of performance and confidence changes between T1 and T2. Male students tended to report higher confidence levels, while training-related factors positively influenced confidence and performance.

**Conclusion:**

A small but growing body of research suggests that self-monitoring accuracy is not a stable trait but changes over time. Our results suggest an improved self-monitoring accuracy at the delayed retest. Future studies should assess self-monitoring in a more fine-grained way and over a longer period of time, and systematically assess inter-individual differences.

## Introduction

Health-care professionals’ capacity to accurately self-assess areas in need of improvement is a fundamental prerequisite in various models of life-long learning and continuous education [1,2,3]. Yet, humans are renowned for their tendency to inaccurately judge their competencies, particularly in areas unfamiliar to them [1,4,5,6], resulting in potentially grave consequences such as diagnostic errors from overconfidence [7]. These errors have been directly related to prolonged hospital stays and increased mortality [8]. However, instead of posing overarching questions like “How skilled am I as a sonographer?” as is typical for eliciting self-assessment judgements, asking contextually bound questions such as “How confident am I in my current task’s execution?” result in much more informative and accurate judgments for learners and practitioners [4,5,6,9]. This moment-to-moment assessment of one’s performance in a specific task has been termed self-monitoring [9,10,11,12]. Self-monitoring can be operationalized as, for example, the relation between self-reported and expert-rated performance or between confidence in one’s task execution and objective performance measures [13,14,15,16]. Research in this tradition suggests that self-monitoring accuracy appears to be greater in high-performing individuals compared to their low-performing counterparts [13]. Also, there is evidence that self-monitoring accuracy exhibits context-specific characteristics, where the difficulty of a particular case might predict students’ self-monitoring accuracy better than their overall proficiency level [14]. Furthermore, it has been shown that feedback can enhance self-monitoring accuracy in immediate follow-up tests [17,18].

Despite these studies advocating a more pertinent conception of self-assessment for clinical practice and education, our understanding of how self-monitoring evolves over time remains limited due to a lack of longitudinal studies. What is more, the current body of research investigating self-monitoring accuracy over time yields a mix of findings. Three studies (two longitudinal, one cross-sectional) indicate that the accuracy of self-monitoring tends to remain relatively stable throughout undergraduate medical education [15,19,20]. Another longitudinal study, however, indicates a decline in self-monitoring accuracy over time [21]. In this study, medical students were tasked with categorizing a set of 50 radiographs as either normal or fractured, accompanied by indicating their level of certainty using “definitely” or “probably.” Subsequent post-tests revealed that diagnostic accuracy correlated with choosing “definitely” over “probably.” However, this correlation diminished after a two-week interval. The observed interaction was attributed to a decline in performance after the two-week period, while the level of certainty in diagnoses remained unchanged.

While Pusic et al. [21] assessed short-term trajectories of self-monitoring, studies investigating long-term trajectories are lacking. Yet, understanding how self-monitoring evolves over longer periods of time, and identifying the factors that shape confidence, could enhance our ability to support students and practitioners in maintaining and improving their self-monitoring skills. For example, if factors such as practice frequency or teaching type would be identified as relevant, instructors could design learning experiences that foster these factors; further, if educators better understood the long-term trajectories of self-monitoring, they could time their support, interventions, or feedback more effectively. Hence, we set out to investigate how self-monitoring accuracy for a complex clinical skill, changes in medical students in the long-term and how training-related factors might affect those changes.

To accomplish this, we designed our retention study in a manner similar to that of Pusic et al. [21], while introducing two key distinctions:

First, in contrast to the task of interpreting radiographs used by Pusic et al. [21], we opted for a more complex clinical task, namely an intricate ultrasound assessment. This task involved not only the interpretation of images but also their generation and allowed for a wide spectrum of potential diagnoses. This contrasts with the binary diagnostic task of identifying normal or abnormal conditions based on ankle radiographs.

Second, between initial and post-test 6 months later, students were allowed to train in a self-directed manner. Unlike Pusic et al.’s study [21], where only the initial and follow-up tests were conducted, our analyses also take into account a number of control variables that might influence performance and/or confidence.

## Methods

### Design

The present study is an observational sub-study of the SIGNATURE randomized controlled trial [22]. In brief, SIGNATURE compared the effect of peer instruction versus faculty teaching of abdominal ultrasound on medical students’ ultrasound performance. Performance was assessed immediately and six months after instruction in objective structured clinical examinations (OSCEs). 152 students participated in the trial. Peer-instructed students performed significantly better than faculty-instructed students immediately post-instruction and at six-month follow-up (all p < 0.001). However, the main analysis did not assess students’ self-monitoring, nor the change in self-monitoring over time, which is the purpose of this secondary analysis [22].

### Subjects

From September 2019 to December 2020, 152 students from three medical schools in Switzerland were enrolled in the SIGNATURE trial. Students were included if they were willing to pay the course fee, signed the study agreement and informed consent and completed a baseline questionnaire. Students with more than 5 hours of previous ultrasound training were excluded. 141 students completed the course and the immediate post-course OSCE and were included in the present sub-study. The study protocol was submitted to the Ethics Committee of the canton of Bern for review and deemed exempt from full ethical review (Req-2019-00537).

### Data collection

Performance in abdominal ultrasound was assessed with the Students’ Ultrasound Skill Assessment at the end of the course (T1) and after six-month follow-up (T2). The assessment consisted of 12 OSCE stations where students assessed different organs with ultrasound (thyroid gland, retroperitoneum with aorta, pancreas, right liver lobe, liver, hepatic vein star, vena portae, gall bladder, right kidney, left kidney, spleen, and bladder). Each student was randomly assigned to a set of 6 out of the 12 OSCE stations. At T2, students were examined at two stations that they had performed at their OSCE at T1 and at four stations that were not part of their OSCE at T1. The rationale was to allow for a direct analysis of test-retest effects on performance measures and at the same time sample all students across the full spectrum of the course content. At each of the OSCE stations, different skills such as transducer handling, patient instruction, examination, image explanation, theory and overall performance were assessed using a previously validated checklist [23] by an expert assessor, certified in abdominal ultrasound. The sum score at each OSCE station ranged from 0–50 points.

After completing each OSCE station, students were asked how confident they were that they had performed well in the task, using a Likert-scale ranging from 1 (not sure at all) to 5 (absolutely sure). At T2, students were asked to self-report additional ultrasound training and practice since T1 (in hours). Additionally, each student filled in a baseline questionnaire that collected demographic information together with data on experience and previous ultrasonography training (in hours) and a German version of the 3-item Cognitive Reflection Test (CRT), scored as the number of correct responses (0–3) [24,25]. The CRT poses problems that tend to trigger an immediate, but incorrect response. In order to solve such a problem correctly, participants have to inhibit and overwrite this spontaneous response [26]. Since its first publication, the CRT has been used in a wide range of studiesshowing good reliability, and substantial correlations to a number of indicators of faulty or biased reasoning have been found [26,27,28].

### Statistical analysis

We analysed the data in two steps. First, we calculated descriptive statistics and a series of univariate analyses. In the second step, we estimated multivariate mixed models.

In the first step, we used counts and proportions or median and quartiles to describe baseline characteristics as appropriate. Furthermore, to assess changes in OSCE performance and confidence over time, we averaged the OSCE ratings and confidence of the six completed stations for each student at T1 and T2, respectively, and performed a Wilcoxon signed-rank test.

In the second step, we used mixed effects models to identify factors related to OSCE performance and confidence. We estimated two independent models: One with confidence and one with performance as the dependent variable. Both models had an identical random effects structure. Specifically, variance components were estimated for subjects (students), OSCE station, and raters (examiners) as random effects to account for the nested structure of the data.

For both models, fixed effects were age, gender (female vs. male), semester (5 vs. 7), hours of previous ultrasound training (0–5), self-reported hours of additional ultrasound training after T1, cognitive reflection test score (0–3), order of OSCE stations (1^st^–6^th^), mode of instruction (peer vs. faculty), and time of OSCE (T1 vs. T2) as fixed factors.

In the model for performance as dependent variable, we additionally added confidence level (1–5) as fixed effect and its interaction with time of assessment. Model assumptions were assessed visually using diagnostic plots. All statistical analyses were performed using statistical software R version 4.0.3. Mixed models were estimated using the R package lme4 [29].

## Results

### Descriptive statistics and univariate analyses

**Figure 1:**
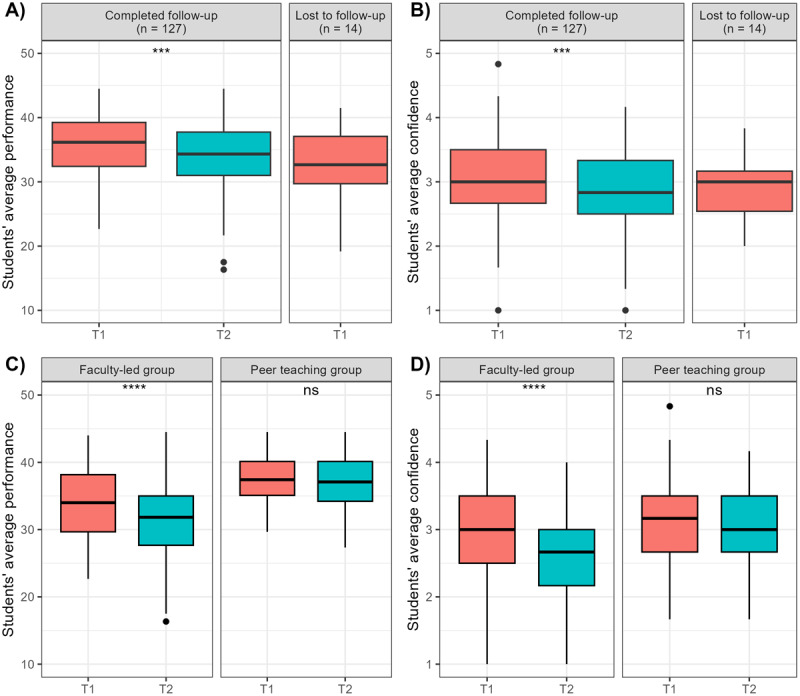
Students’ average performance and confidence at time points T1 and T2, **(A)** average performance and **(B)** confidence for completed follow-up vs. lost to follow-up, **(C)** average performance and **(D)** confidence in the faculty-led group vs. peer-teaching group.

Baseline characteristics of the 141 included medical students (65% female) are shown in Table 1. Median age (interquartile range; IQR) was 22 (21 to 23) years and all students were either in their fifth (63%) or seventh (37%) semester. The majority (60%) answered all three questions of the cognitive reflection test correctly. Fourteen participants did not take part in the OSCE at T2 and were considered lost-to-follow-up. As can be seen in Figure 1, performance of participants that were lost to follow-up was non-significantly lower with a of 32.7 (29.71–37.1) points compared to participants that completed the study, while confidence was comparable with a median confidence level of 3 (2.5–3.2).

**Table 1:** Characteristics of study participants.


CHARACTERISTIC	MEDIAN (IQR); N (%) (N = 141)

Age (years)	22.00 (21.00, 23.00)

Female gender	92 (65%)

Semester	

5	89 (63%)

7	52 (37%)

Previous ultrasound-education (hours)	

0	68 (48%)

1–2	50 (35%)

3–5	23 (16%)

Cognitive Reflection Test (number of correct answers)	

0	8 (5.7%)

1	14 (9.9%)

2	35 (25%)

3	84 (60%)


**Figure A1:**
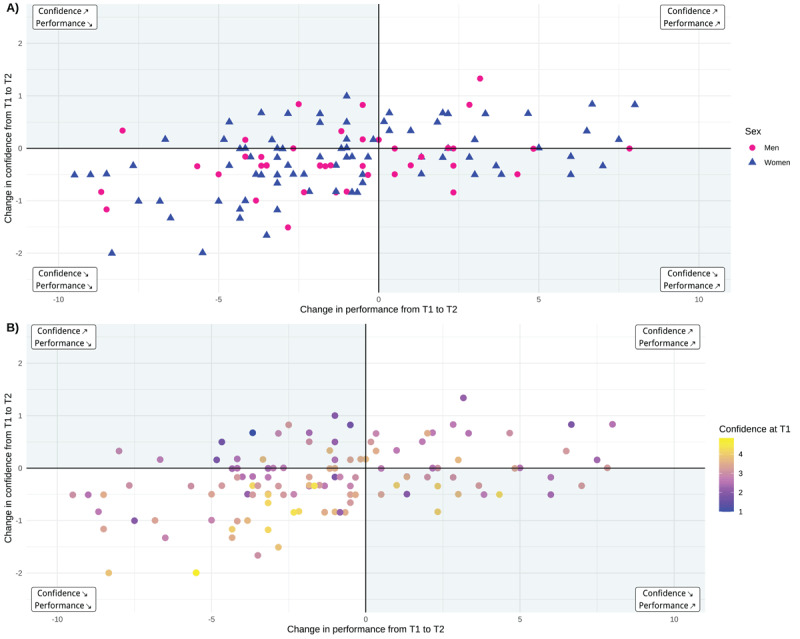
Changes in confidence and changes in performance per participant from time point T1 to T2, **(A)** by gender, **(B)** by confidence level at T1.

In a univariate analysis, the performance score averaged across all six OSCE stations for each of the remaining 127 participants was significantly higher at T1 with a median (IQR) of 36.0 (31.7–39.2) points compared to T2 with 34.3 (31.0–37.8) out of 50 points (Figure 1A; Wilcoxon signed-rank test: V = 5652.5, Z = –4.02, p < .001, r = .36). Likewise, average confidence significantly decreased from a median of 3.0 (2.7–3.5) at T1 to 2.8 (2.5–3.3) at T2 (Figure 1B; Wilcoxon signed-rank test: V = 1831.5, Z = –3.76, p < .001, r = .36). Changes between T1 and T2 varied in direction and magnitude between participants (Figure A1 in Appendix).

### Linear mixed model: Time-based differences in association between confidence and performance

The main outcome of interest was a possible difference in the relation between confidence and performance at T1 as compared to T2, with the corresponding results presented in Table 2 (for a presentation of all results, see Appendix Table A1).

**Table 2:** Results from two linear mixed models with the dependent variable performance, only the fixed effects that are of main interest for this study are printed. Detailed results for control variables are given in Table A1.


*DUMMY MODEL FORMULA:* PERFORMANCE ~ T2 + CONFIDENCE + T2 * CONFIDENCE + CONTROL VARIABLES + RANDOM EFFECTS	DEPENDENT VARIABLE: PERFORMANCE [0–50]			

	*ESTIMATES*	*std. Beta*	*95% CI*	*p*

T2	–2.59	–0.09	–4.45, –0.74	**0.006**

confidence	3.17	0.40	2.78, 3.56	**<0.001**

T2 * confidence	0.62	0.08	0.08, 1.16	**0.023**


*Note*. T2 is the OSCE at 6-month follow-up; CI, confidence interval; p-values p < .050 written in bold; control variables were: Age; Female gender; study semester; Previous US education; Additional US training after T1; Peer teaching vs traditional group; Cognitive Reflection Test; OSCE station in order; for the full results please refer to the supplement.

**Figure 2:**
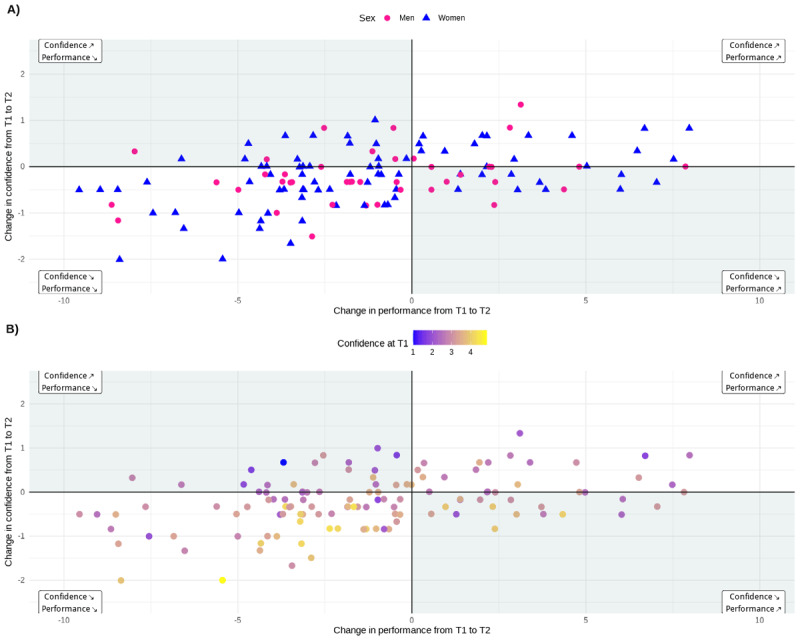
Students’ individual confidence over their individual performance at time points T1 and T2.

The self-reported confidence level was positively associated with the performance as rated by the examiners. Taking a set of control variables into account, we found that confidence was significantly associated with performance, with a moderate-to-large effect size (std Beta = 0.40, p < .001). Furthermore, there was a small effect for decrease in performance from T1 to T2 (std. Beta = –0.09, p = .006, cf. Table 2). Finally, we found a positive, small effect for the interaction between time point and confidence (std. Beta = 0.08, 0.023). This effect suggests that students were better aligned in self-evaluating their performance on the second occasion compared to the first occasion. In a post-hoc analysis, we found a stronger correlation between the average confidence rating and average performance at T2 (Pearson correlation coefficient = .69) compared to T1 (.5, *p* = .024), illustrated in Figure 2. Note that these correlations are not adjusted for control-variables and thus are overestimating the effects as compared to the mixed effects model in Table 2. Still, in summary our results suggest that self-monitoring improved over time.

As for the control variables included, we found that performance was significantly higher in seventh semester students compared to fifth semester students and in students who participated in the peer teaching group and/or had additional practice hours after T1.

We furthermore ran a linear mixed effects model for confidence as a dependent variable (Appendix Table A1, right column) showing that male students gave numerically higher confidence ratings than female students (std. Beta = –0.37, p < .001), although performance did not differ between genders (std. Beta = 0.07; p = .35). Training-related factors (previous ultrasound training, peer teaching, additional training after T1) were positively associated with higher confidence.

### Sub-analyses

As reported in the primary publication of this trial [22], only the faculty-led group experienced a decline in performance from T1 to T2 (Figure 1C). Interestingly, the same applied to the decline in confidence between T1 and T2: while a significant decline can be observed in the faculty-led group of learners, confidence in the peer-teaching group remained at similar levels, mirroring the stable performance over time in this group (Figure 1D).

## Discussion

### Summary of findings

With our retention study, we aimed to shed light on the changes of self-monitoring accuracy in medical education over an extended time span of six months. In line with previous studies [30], we observed a skill decay over time. Yet, unlike in the only directly comparable past study [21], which reported that confidence remained aligned with the initial peak level of performance over a two-week retention interval, suggesting *decreased* self-monitoring over time, we observed the opposite: in the majority of students, self-reported confidence decreased in some proportion to their skill decline over the follow-up period, suggesting an *improved* self-monitoring accuracy at the delayed retest six months after the first assessment. Notably, we also observed relevant inter-individual variability in self-monitoring changes, highlighting a potential target for systematic instructional interventions.

### Comparison with previous research

Methodological differences between ours and Pusic et al.’s study [21] are likely part of the explanation for why these differences between studies occurred. First, follow-up intervals between both studies were substantially different, from two weeks in Pusic et al.’s study to 6 months in ours. It may very well be possible that skill decay happens faster than a reduction in confidence, leading to substantial overconfidence after shorter periods, as observed by Pusic et al., but levelling off into adequate (or even improved) self-monitoring later. To study this hypothesis, future studies may strive to track changes of self-monitoring in a more fine-grained way and over a longer period of time by incorporating several regular intermediate assessments, for example, by analysing data from progress tests that periodically formatively assess clinical knowledge and assess related confidence [15,20]. Moreover, future research may address the possible instructional implications of this difference in findings as it may suggest that the optimal timing for bolus refresher education is when the spacing between repetitions is long enough to allow a level of forgetting that reintroduces a sense of uncertainty in one’s abilities [31,32]. Furthermore, a large body of previous research explores the effect of instructional design on performance changes (e.g. of spaced versus blocked practice). The study for which we conducted a secondary analysis here belongs to that area of research, as it investigated the effect of faculty versus peer lead instruction on performance and found peer led instruction to result in better long term performance retention. This effect is associated with higher confidence ratings in the follow up measurement in the peer instructed group. It is unclear whether the higher confidence at follow up is an effect of better performance or an effect of the instructional method, which implies that future research should specifically explore the role of instructional format on confidence and self-monitoring.

As we did not provide feedback on performance nor self-monitoring accuracy in this study, it is left for future research to investigate whether regular task-specific feedback on self-monitoring accuracy may enhance self-monitoring over time as is indicated by research into self-assessment [33,34], for example, by triggering an adequate level of further self-directed training. Such feedback could be integrated in regular progress test reports, ideally together with advice on how to practice topics for which students lack accurate self-monitoring.

Second, Pusic et al. [21] used dichotomous measures for performance (fractured versus non-fractured, being either a correct or incorrect reading of the test X-rays) and confidence (definitely versus probably). In contrast, our study used a continuous measure of performance (0–50) and a more fine-grained Likert-like confidence scale (1–5). As a consequence, our study may have larger power to assess changes in both measures and may identify more subtle associations between them and with confounding variables.

Third, both studies differ substantially in the tasks assessed and thus the cues available for insights into one’s performance. The notion that ‘cues’ are used to guide self-monitoring is drawn from the cue-utilisation framework put forward by Koriat [35,36,37]. His work demonstrated that humans cannot directly judge the quality of their cognitive operations, but utilise cues such as the perceived fluency of processing to monitor whether they have understood and learned the material. Some cues, such as the speed with which a solution comes to mind or mental effort, can be ‘diagnostic’ of actual performance (i.e. provide meaningful information regarding the likelihood of success), whereas others can be misleading [28,38,39]. Students in the Pusic et al. study were asked to classify pathologies in X-rays as fractured or non-fractured and rate their confidence as definitely or probably. Besides task-independent cues such as their prior experience [40], the only metacognitive cues available to them to judge their performance on this task were the speed with which they could identify the pathology and the correspondence of the pathology (or normality) to previously memorized examples. In our study, students were asked to visualize a certain anatomical structure with an ultrasound probe, capture an image, explain it to the patient and assessor, and answer predefined questions on it. This arguably more complex task provides the same plus many additional metacognitive cues to self-monitor one’s performance, such as how easily the structure was found, how good the image generated was, as well as potential non-verbal patient and assessor cues, and the ease with which answers to questions came to mind. This multitude of metacognitive cues may have resulted in higher self-monitoring accuracy.

Our analyses also revealed other factors being associated with performance and/or confidence, corroborating previous findings: We found male and more advanced students giving higher confidence ratings than their female or less advanced colleagues, independent of actual performance, a finding in line with previous research [14,15]. This finding reinforces the analytical approach to assess self-monitoring accuracy on an individual level rather than on aggregated data [19,41]. Moreover, training-related factors (e.g., peer teaching as opposed to faculty-led courses, additional training) were also related to higher performance and confidence.

In summary, a small but growing body of research suggests that self-monitoring accuracy is not a stable trait but changes over time. This finding opens up the opportunity to identify predictive cues of one’s performance, teach them to students, and evaluate the effect of such teaching on self-monitoring accuracy to ultimately achieve more accurate self-monitoring.

### Limitations and future directions

Our study comes with some limitations concerning its generalizability to other than student populations and tasks other than ultrasound. Particularly, given the “generality of context specificity” of one’s performance [42], further research is needed to examine whether self-monitoring accuracy is similarly context-specific. Another limitation can be seen in our way of assessing confidence, which relied on a 5-point Likert scale. Although this was a more fine-grained scale than those used in previous studies [15,21], it did not align directly with the performance ratings, which were measured on a 0–50-point scale. Moreover, there was another difference in the granularity of measurements: performance was assessed across multiple specific skills per OSCE, whereas confidence was measured using a single, general question per OSCE station. This mismatch may have complicated the confidence judgment for learners relative to specific aspects of their performance. Future studies may consider employing more granular and task-specific measures of confidence and explore how different response formats impact self-monitoring results [41,43]. Another possible limitation is that our lost-to-follow-up students had lower performance scores, while confidence levels were comparable. This pattern suggests a potential attrition bias as the final sample overrepresents higher-performance students. Results may thus not fully generalize to lower-performing students. Concerning the factors we included in our analyses, training hours were assessed retrospectively over a six-month period and relied on self-report, which may be prone to recall bias, rounding, or social desirability effects. Such inaccuracies could have introduced both random and systematic error; therefore, results involving training hours should be interpreted cautiously. Last, all analyses reported here were explorative without adjusting for multiple testing.

## Conclusion

In summary, our retention study suggests that, at least in complex tasks such as abdominal ultrasound that provide some metacognitive cues to inform confidence judgements, self-monitoring accuracy does increase over an extended period of time. Further research is needed to investigate the role of regular feedback and the context specificity of this effect in order to be able to better support the next generation of healthcare professionals for safe practice.

## Appendix

**Table A1:** Full results from two linear mixed models with the dependent variables performance and confidence.

***FIXED EFFECTS***	MODEL FOR PERFORMANCE [0–50]				MODEL FOR CONFIDENCE [1–5]			
	
	*ESTIMATES*	*std. Beta*	*95% CI*	*p*	*ESTIMATES*	*std. Beta*	*95% CI*	*p*

Age	–0.15	–0.04	–0.42, 0.11	0.263	–0.02	–0.04	–0.06, 0.02	0.321

Female gender (Ref. Male)	0.57	0.07	–0.62, 1.75	0.35	–0.37	–0.35	–0.54, –0.19	**<0.001**

7th study semester (Ref. 5)	1.86	0.22	0.02, 3.70	**0.047**	0.29	0.27	–0.01, 0.58	0.060

Previous US education [h]	0.25	0.05	–0.14, 0.65	0.205	0.06	0.08	0.00, 0.12	**0.047**

Additional US training after T1 [h]	0.24	0.1	0.09, 0.40	**0.003**	0.02	0.07	–0.00, 0.05	0.077

Peer teaching group (Ref. faculty-led group)	3.87	0.46	2.75, 4.99	**<0.001**	0.32	0.30	0.15, 0.48	**<0.001**

Cognitive Reflection Test [0–3 points]	0.29	0.03	–0.34, 0.92	0.372	–0.04	–0.04	–0.14, 0.05	0.390

OSCE station in order [1–6]	0.21	0.04	0.06, 0.36	**0.006**	0.04	0.06	0.01, 0.06	**0.002**

T2 (Ref. T1)	–2.59	–0.09	–4.45, –0.74	**0.006**	0.06	0.06	–0.11, 0.23	0.492

Confidence level [1–5]	3.17	0.4	2.78, 3.56	**<0.001**				

Confidence level [1–5] * T2	0.62	0.08	0.08, 1.16	**0.023**				

***Random intercepts***								

σ^2^	26.9				0.70			

τ_00 students_	8.44				0.18			

τ_00 examiners_	3.45				0.10			

τ_00 osce stations_	4.5				0.11			

N_students_	141				141			

N_osce stations_	12				12			

N_examiners_	29				29			

US, Ultrasonography; T1, OSCE at baseline; T2, OSCE at 6-month follow-up; σ^2^, residual variance; τ_00_, variance random intercept; N, number of groups; CI, confidence interval; p-values p < .050 written in bold.
